# Use of the novel contact heat evoked potential stimulator (CHEPS) for the assessment of small fibre neuropathy: correlations with skin flare responses and intra-epidermal nerve fibre counts

**DOI:** 10.1186/1471-2377-7-21

**Published:** 2007-08-03

**Authors:** Duncan D Atherton, Paul Facer, Katherine M Roberts, V Peter Misra, Boris A Chizh, Chas Bountra, Praveen Anand

**Affiliations:** 1 Peripheral Neuropathy Unit, Hammersmith Hospital and Imperial College London, UK; 2 Clinical Pharmacology and Discovery Medicine, GlaxoSmithKline, Cambridge, UK; 3 Neurology and GI CEDD, GlaxoSmithKline, Harlow, UK

## Abstract

**Background:**

The Contact Heat Evoked Potential Stimulator (CHEPS) rapidly stimulates cutaneous small nerve fibres, and resulting evoked potentials can be recorded from the scalp. We have studied patients with symptoms of sensory neuropathy and controls using CHEPS, and validated the findings using other objective measures of small nerve fibres i.e. the histamine-induced skin flare response and intra-epidermal fibres (IEF), and also quantitative sensory testing (QST), a subjective measure.

**Methods:**

In patients with symptoms of sensory neuropathy (n = 41) and healthy controls (n = 9) we performed clinical examination, QST (monofilament, vibration and thermal perception thresholds), nerve conduction studies, histamine-induced skin flares and CHEPS. Skin punch biopsies were immunostained using standard ABC immunoperoxidase for the nerve marker PGP 9.5 or the heat and capsaicin receptor TRPV1. Immunoreactive IEF were counted per length of tissue section and epidermal thickness recorded.

**Results:**

Amplitudes of Aδ evoked potentials (μV) following face, arm or leg stimulation were reduced in patients (e.g. for the leg: mean ± SEM – controls 11.7 ± 1.95, patients 3.63 ± 0.85, p = 0.0032). Patients showed reduced leg skin flare responses, which correlated with Aδ amplitudes (r_s _= 0.40, p = 0.010). In patient leg skin biopsies, PGP 9.5- and TRPV1-immunoreactive IEF were reduced and correlated with Aδ amplitudes (PGP 9.5, r_s _= 0.51, p = 0.0006; TRPV1, r_s _= 0.48, p = 0.0012).

**Conclusion:**

CHEPS appears a sensitive measure, with abnormalities observed in some symptomatic patients who did not have significant IEF loss and/or QST abnormalities. Some of the latter patients may have early small fibre dysfunction or ion channelopathy. CHEPS provides a clinically practical, non-invasive and objective measure, and can be a useful additional tool for the assessment of sensory small fibre neuropathy. Although further evaluation is required, the technique shows potential clinical utility to differentiate neuropathy from other chronic pain states, and provide a biomarker for analgesic development.

## Background

Small fibre neuropathy is commonly caused by diabetes, and also a number of other conditions, including amyloid, HIV, neurotoxin exposure and hereditary and idiopathic diseases. The presenting features may include pain, numbness, or hypersensitivity in the peripheral limb, usually the feet, progressing proximally in a length-dependent fashion, with or without autonomic symptoms. Diagnosis of sensory peripheral neuropathy is usually by nerve conduction studies, which assess mainly large sensory fibre dysfunction. To assess small sensory fibre function, methods such as thermal quantitative sensory testing (QST) are commonly applied.

The Contact Heat Evoked Potential Stimulator (CHEPS) technology utilizes rapidly delivered heat pulses with adjustable peak temperatures to stimulate the differential warm/heat thresholds of receptors expressed by Aδ and C fibres. The resulting evoked potentials can be recorded and measured. CHEPS has been used to selectively excite Aδ and C fibres in human volunteer glabrous and hairy skin [1]. Contact heat evoked potentials have also been studied previously in volunteer models, where reliable and quantifiable evoked potentials were produced, with consistent Aδ peak latencies and amplitudes particularly in the Cz component, and with significant correlation to pain intensity scores [2,3]. Volunteer models have been used to illustrate the reproducibility of cerebral responses (blood oxygen level dependent fMRI changes) and subjective responses (pain scores) to different intensities of CHEPS stimuli [4]. A strong similarity between CHEPS evoked potentials and Laser Evoked Potentials has been described [5]; CHEPS offers the advantages of being easier to use in the clinic, does not require eye protection, and reduces risk of inducing burns or erythema. It also allows for repetitive stimulation or 'wind-up'. CHEPS thus offers an additional clinical tool for the assessment of small sensory nerve fibre function.

We have studied 41 patients with a clinical diagnosis of sensory fibre neuropathy, focussing on CHEPS in comparison with other objective tests such as histamine-induced skin flares and intra-epidermal fibre (IEF) counts in skin biopsies. The flare following intradermal histamine injection (axon-reflex vasodilatation) is mediated by mainly by C fibres, which release neuroeffectors such as calcitonin gene-related peptide (CGRP) and substance P from skin terminals, with consequent vasodilatation and extravasation. The flare is a component of the classical Lewis' triple response, and can be used for the assessment of small sensory fibre loss or dysfunction [6-10]. Intra-epidermal nerve fibre density on skin biopsy has been described previously as a valid objective method for quantitative assessment of small fibre neuropathy, and is regarded as the preferred test [11-13]. We have included TRPV1 nerve fibre counts in this study, as CHEPS activates cutaneous nerve terminals which express TRPV1, the heat and capsaicin receptor, in the protocol we have used.

## Methods

### Patients

Forty one patients with a clinical diagnosis of sensory neuropathy [mean age years 55 (range 36–77); 25 female] were studied. All patients were seen in the neuropathy clinic at Hammersmith Hospital. All procedures were undertaken with full patient written consent and approval of the Hammersmith Research Ethics Committee. The mean duration of symptoms was 4.7 years (range 4 months – 17 years). The majority of patients (n = 29) had idiopathic neuropathy; of the remaining 12, one had glucose intolerance detected 3 years after onset of symptoms, three patients were early diabetics, two had treated vitamin B12 deficiency, two a previous history of statin use, two a history of moderate alcohol intake, one a recent history of chemotherapy, and one a history of vasculitis. Thirty four patients had painful neuropathy, and most were taking treatment at the time of review – twelve were taking Gabapentin, five Pregabalin, six Amitryptyline and a single prescription each of Paroxetine, Mexiletine, Clonazepam, Levetiracetam. However, no patients experienced sedation at the time of the study, and future systematic studies are indicated to determine the effect of medications. The mean age of control subjects was 43.6 years (range 21 to 70).

Full history, clinical examination, contact heat evoked potentials, skin flare response to histamine, and skin punch biopsies were performed in all patients. Nerve conduction studies of the sural and peroneal nerve were carried out, although not all at the same institution. Values less than 5 μV amplitude and 40 m/s conduction velocity were considered abnormal for the antidromic sural sensory action potential and values less than 3 mV amplitude and 40 m/s conduction velocity were considered abnormal for the common peroneal nerve (compound muscle action potential from extensor digitorum brevis). Quantitative Sensory Tests (QST) were also performed, including monofilament, vibration and thermal perception thresholds, as described previously [14-16]. Thresholds for touch were measured using Semmes-Weinstein hairs (made by A. Ainsworth, University College London, UK). The number of the hair with the lowest force reliably detected by the patient (at least 3 out of 5 trials) on the dorsum of the most affected big toe was recorded. The 'abnormal' value was designated as >No. 3 monofilament (0.0479 g). Vibration perception thresholds were measured using a biothesiometer (Biomedical instrument Co., Newbury, OH, USA) placed on the metatarsophalangeal joint of the big toe. Three ascending and three descending trials were carried out, and the mean value obtained. The vibration threshold was expressed in volts; values >12 V were considered 'abnormal'. Thermal perception thresholds were performed as previously described using Thermotest (Somedic, Stockholm, Sweden). A 15 × 25 mm thermode was used and thermal thresholds determined at the lateral calf for warm and cool perception from a baseline temperature of 30°C, with a change in temperature of 1°C/sec. The mean of three consecutive tests for each modality was recorded. Values >6.4°C for warm sensation, >2.3°C for cool sensation were considered abnormal.

### Contact heat evoked potentials

We used a Contact Heat Evoked Potentials Stimulator (CHEPS), (Medoc Ltd, Ramat Yishai, Israel) with a thermode area of 572.5 mm^2^, and a heating thermo-foil (Minco Products, Inc., Minneapolis, MN) covered with a 25 μm layer of thermo-conductive plastic (Kapton^®^, thermal conductivity at 23°C of 0.1 – 0.35 W/m/K). The thermode heating rate was 70°C/s and the cooling rate 40°C/s. The baseline temperature was 32°C, destination temperature 51°C, and stimulus interval 7 seconds. In this paper we have focused on Aδ amplitudes, as they provided the most robust signal.

Evoked potentials were recorded from six midline electrodes (Fz, FCz, Cz, CPz, Pz, POz), using a similar protocol to that described previously [1]. The ground electrode was placed on the left temporal region. Each recording epoch of 2800 ms included a period of 200 ms baseline before stimulus onset. A low cut off filter with a time constant of 1.06103 and a frequency of 0.15 Hz and a high cut off filter of 100 Hz were applied. The impedance from all electrodes was maintained below 5 Ω and the electroencephalogram (EEG) recorded and digitized at a sampling rate of 500 Hz. We report data from the vertex (FCz) position for Aδ potentials following skin stimulation.

The recorded EEG data were analysed using Vision Analyser^© ^Version 1.05.001 (Brain Products, Ltd., London UK). Recordings of eye movement artefacts from supra- and infra-orbital electrodes were subtracted from the cerebral trace, and the mean of resulting sweeps obtained. Within each waveform, the latency was measured from the first definitive negative peak (N2), and the amplitude measured peak-to-peak (N2 to P2). The sweep speed used when the waveform was measured was 225 ms per cm.

### Histamine skin flare

Histamine (0.03 ml of 1 mg/ml. Martindale Pharmaceuticals, Romford, UK) was injected intra-dermally into the lateral calf of patients (n = 41) and controls, as described previously [14], and the area of flare measured after 10 minutes by scanning Laser Doppler imaging (Moor Instruments Ltd, Axminster UK). On the flux image the region of interest (defined by the edge of the flare) was drawn, and the area measured using the Moor Laser Doppler Imager (LDI) version 3.11 software [10].

### Skin Biopsies

Two 3 mm diameter skin punch biopsies were collected under local anaesthesia from the leg (lateral calf) of patients (n = 41) and control subjects (n = 9) for immunohistology. Skin biopsy was taken from the same area as was used for CHEPS stimulation.

### Immunohistology

The immunohistochemical methods and antibodies used have been reported by us previously [7,17,18]. One of the two skin biopsies was snap frozen and stored at -70°C, and the other immersed in fixative (modified Zamboni's fluid – 2% formalin; 0.01 M phosphate buffer; 15% saturated picric acid pH 7.2), then washed in phosphate buffered saline (PBS; 0.1 M phosphate; 0.9% w/v saline; pH 7.3) containing 15% w/v sucrose for an hour, before snap freezing in embedding medium (Tissue-Tek OCT, RA Lamb, Sussex, U.K.).

Frozen sections (10 μm) of pre-fixed and unfixed samples were collected onto poly-L-lysine-coated (Sigma, Poole, Dorset UK) glass slides. Unfixed sections were then post-fixed in freshly prepared, 4% w/v paraformaldehyde in PBS. All sections were washed in PBS, and endogenous peroxidase blocked by incubation in 0.3% w/v hydrogen peroxide in methanol. After rehydration, the tissue sections were incubated overnight with primary antibodies to the structural nerve marker PGP 9.5 (Ultraclone Ltd, Isle of Wight, UK; 1/50,000; for immersion fixed tissue sections) or the heat and capsaicin receptor TRPV1 (GlaxoSmithKline, Harlow, UK; 1/5,000, for post-fixed tissue sections). Immersion fixed sections were used for optimal staining of PGP 9.5, and frozen, post-fixed sections were used for optimal immunostaining of TRPV1. Controls included omission of primary antibodies, or their replacement with pre-immune serum. Sites of antibody attachment were revealed using a nickel-enhanced, immunoperoxidase method (avidin-biotin complex – ABC elite; Vector Laboratories, High Wycombe, Bucks., U.K.). Nuclei were counterstained with 0.1% w/v aqueous neutral red. Intra-epidermal and sub-epidermal fibres immunoreactive for PGP 9.5 and TRPV1 were counted in a blinded manner, from up 4 – 5 sections from each biopsy, and the length of the epidermis measured using a calibrated microscope eyepiece graticule. Results were expressed as fibres per mm length of epidermis.

Epidermal thickness was quantified from post-fixed frozen tissue sections (10 μm). One section was randomly selected for each control or patient, and epidermal thickness measured at three points along the length of the epidermis using a calibrated microscope, eyepiece graticule and a × 40 objective. Epidermal thickness was defined as the distance between the epidermal-dermal junction and the outermost stratum corneum.

### Statistical Analysis

Graphs were created and statistical tests (Mann Whitney and Spearman correlation coefficients) were performed using GraphPad Prism version 3.02 for Windows, (GraphPad Software, San Diego California USA).

## Results

### Clinical examination and QSTs

Thirty four patients complained of pain related symptoms, of which "burning" was the most frequent description (n = 15). The spontaneous or on-going pain score using a numeric rating scale (NRS, where 0 is no pain and 10 is maximum pain) was 7.4 (range 4.2 – 9). Ten patients complained of numbness in the distal lower limbs, and 2 patients, additionally, in the hands; 10 patients reported lower limb skin hypersensitivity (to either mechanical or thermal stimuli). None of the patients had weakness or muscle wasting. Ten patients had reduced ankle jerks. No patient had abnormality of light touch (cotton wool) sensation. Sixteen patients had reduced pinprick sensation in the distal lower limb. Twenty patients had elevated monofilament perception thresholds. Twenty patients had elevated vibration perception threshold. Analysis of results from patients revealed the mean warm perception threshold as 8.2 ± 0.8°C change and cool perception threshold as 3.6 ± 0.6°C change at the calf. Thermal perception thresholds were within normal limits in 20 patients for warming, and in 21 patients for cooling. Nerve conduction studies showed abnormalities in 7 patients for sural nerve sensory action potentials; all motor studies were normal.

### Contact heat evoked potentials

**Figure 1:**
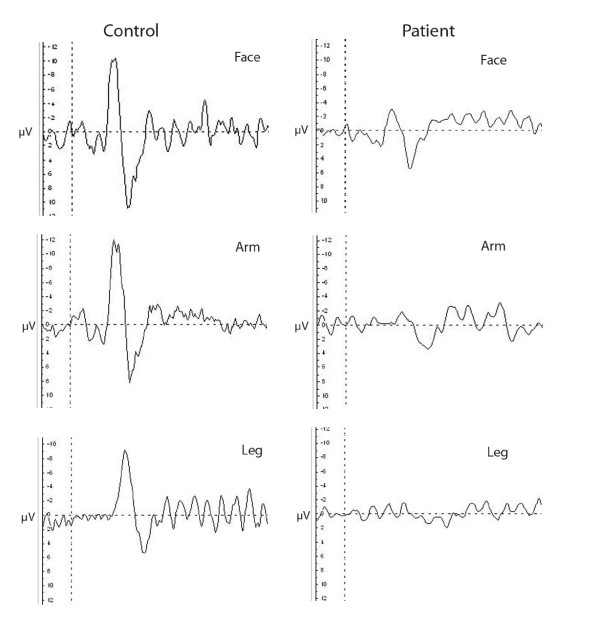
Contact Heat Evoked Potentials (Aδ) from a control subject (left panel) and a patient (right panel) on stimulation of different regions (reported from FCz electrode). Latencies (ms); Control: Face 380, Arm 410, Leg 500; Patient: Face 440, Arm 560, Leg not recordable. Amplitudes (μV); Control: Face 20.86, Arm 20.07, Leg 14.46; Patient: Face 8.45, Arm 5.22, Leg not recordable.

**Figure 2:**
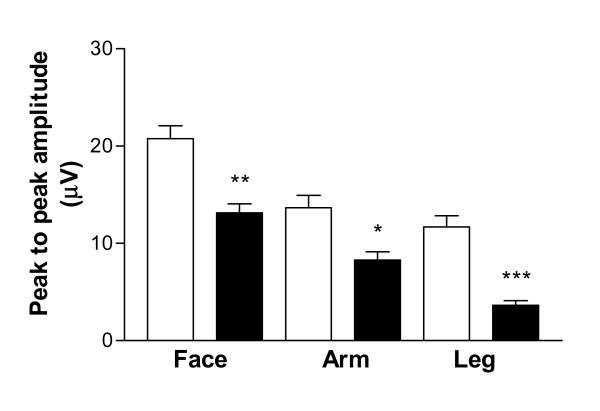
Contact Heat Evoked Potential Aδ amplitudes following stimulation of the face, arm and leg of controls (white bars) and patients (black bars). * = 0.034, ** p = 0.02, *** p = 0.0032.

Examples of an evoked potential trace from a control subject and a patient are shown in Figure 1. Evoked potentials recorded from patients showed reduced Aδ amplitudes compared to controls (Leg Aδ amplitude, mean μV ± SEM, controls 11.7 ± 1.95, patients 3.63 ± 0.85, p = 0.0032; Arm Aδ amplitude, controls 13.67 ± 2.21, patients 8.28 ± 1.48, p = 0.034; Face Aδ amplitude, controls 20.75 ± 2.32, patients 13.14 ± 1.59, p = 0.02: Figure 2). In 24 patients there were no recordable evoked potentials on stimulation of the leg, and in some cases of the arm (n = 13) and face (n = 4). In contrast, evoked potentials were easily recordable in all control subjects from all regions. Sixteen patients with thermal perception thresholds within normal limits had reduced Aδ amplitudes of evoked potentials in the leg (defined as < mean – SEM of controls).

There were no significant differences in latency between patients and controls for face, arm or leg stimulation (Leg Aδ latency, ms, mean ± SEM, controls 525 ± 0.017, patients 509 ± 0.015, p = 0.141; Arm Aδ latency, controls 446 ± 0.009, patients 439 ± 0.020, p = 0.020; Face Aδ latency, controls 397 ± 0.011, patients 423 ± 0.012, p = 0.56). Evoked potential amplitudes from the face, arm and leg were not significantly correlated with age in patients (Face r_s _= -0.2130 p = 0.2056; Arm r_s _= -0.01452 p = 0.9415; Leg r_s _= -0.01228 p = 0.9627). In this study we found no relationship between age and amplitude of evoked responses in controls, but in other studies with larger numbers of controls an inverse relationship with age was obtained (data not shown).

**Figure 3:**
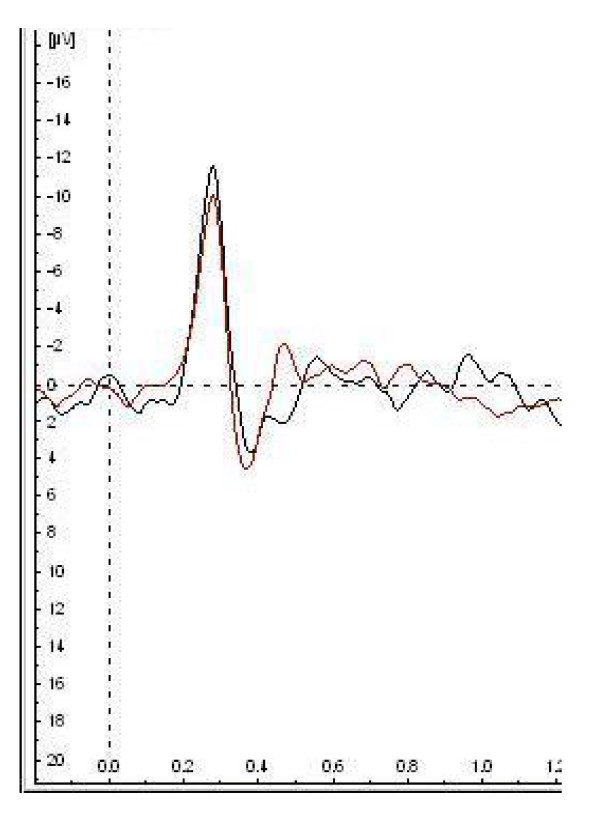
Two superimposed Aδ evoked potentials from a control subject on stimulation of the face, recorded on different occasions.

There was good reproducibility of evoked potential latency and amplitude from the same controls on two occasions (in the morning, and afternoon on the same day) (n = 4); intra-class correlation coefficient: for latency = 0.78, amplitude = 0.86). Figure 3 shows two superimposed Aδ evoked potentials from the face of a control subject recorded on different occasions. We have also shown good reproducibility of control evoked potential amplitude from the arm in a different set of 8 control subjects, with a significant correlation between the repeated amplitudes on two separate days (r_s _= 0.8095, p = 0.0218). Cerebral and subjective responses to different intensities of CHEPS stimulation (sensory detection, pain detection and pain toleration thresholds) have previously been shown to be reproducible across individuals and across multiple sessions [4].

Pain scores (mean ± SEM) from CHEPS application were: Face – controls 7.4 ± 0.4, patients 6.6 ± 0.57; Arm – controls 6.3 ± 0.6, patients 4.3 ± 1.01; Leg – controls 5.0 ± 0.9, patients 3.5 ± 0.76. There was no significant difference in the pain scores between controls and patients for all sites tested (Face p = 0.0825; Arm p = 0.0641; Leg p = 0.1313).

### Histamine-induced flares

Patients showed a reduced mean flare area of response compared to controls (flare response (cm^2^) mean ± SEM; controls 18.14 ± 2.28; patients 12.03 ± 0.90; p = 0.02); there was no difference between flux units in control and patient flares (patient flux mean 444 ± 13.7; control flux 454 ± 46.4; p = 0.61).

### Skin biopsies

**Figure 4:**
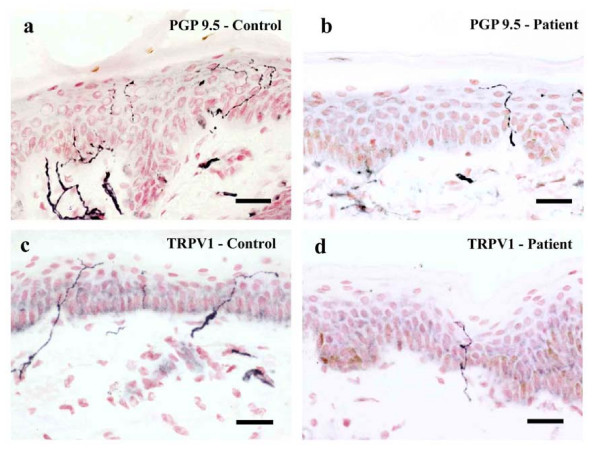
Intra-epidermal fibres for both the nerve marker – (PGP 9.5; a, b) and capsaicin receptor (TRPV1; c, d) are reduced in patient's skin. Scale bar = 50 μm.

Antibodies to both PGP 9.5 and TRPV1 showed immunostaining of nerve fibres in the dermis and sub-epidermis, with fine, branching, intra-epidermal fibres, often extending to the stratum corneum, in control and patient biopsies. The number of intra-epidermal PGP 9.5-immunoreactive fibres per mm was reduced in patient biopsies (mean ± SEM; intra-epidermal: controls, 6.52 ± 1.43 (n = 6); patients, 3.76 ± 0.48 (n = 41); p = 0.027 Figure 4a, b). Intra-epidermal TRPV1-immunoreactive fibres per mm were significantly reduced (mean ± SEM; intra-epidermal: controls, 5.7 ± 1.4 (n = 9); patients, 3.45 ± 0.52 (n = 41); p = 0.04 Figure 4c, d). There was a strong correlation between numbers of nerve fibres immunostaining for PGP 9.5 and TRPV1 (r_s _= 0.6049, p < 0.0001). Prominent swelling of intra- and sub-epidermal fibres was seen in only one patient with both nerve markers. Epidermal thickness measurements revealed a significant reduction in patient biopsies (mean ± SEM, controls 100.1 ± 15 μm (n = 12), patients 73.0 ± 2.8 μm (n = 41), p = 0.0143).

### Correlations of objective tests and contact heat evoked potentials

#### Histamine-induced flares

**Figure 5:**
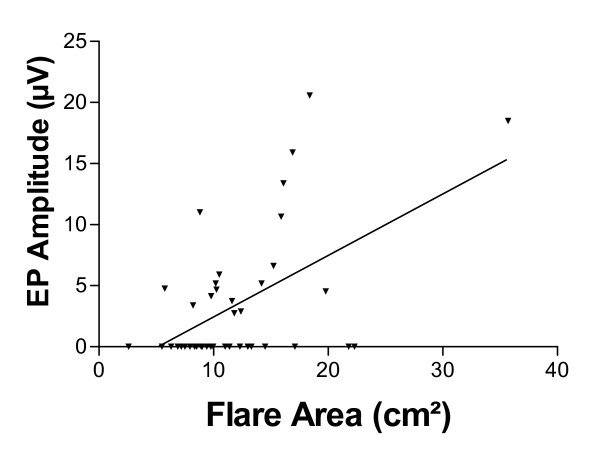
Plot of histamine induced flare area vs. Contact Heat Evoked Potential Aδ amplitudes after leg stimulation.

A positive correlation was demonstrated between flare area and evoked potential Aδ amplitudes (r_s _= 0.40, p = 0.010; Figure 5), and also nerve fibre (PGP 9.5) density (r_s _= 0.44, p = 0.0036), but did not just reach significance between nerve fibre density (TRPV1) and histamine induced skin flare area in the leg (r_s _= 0.300, p = 0.053). There was no statistical correlation between leg flare area and arm evoked potential Aδ amplitudes (r_s _= 0.10, p = 0.55).

#### Skin biopsies

**Figure 6:**
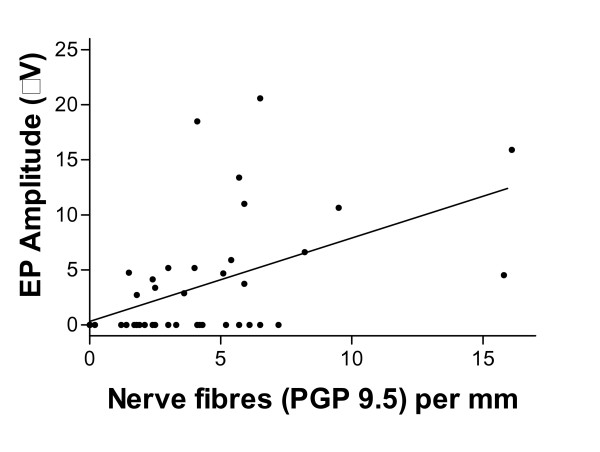
Plot of patient intra-epidermal nerve fibres (PGP 9.5) in skin biopsies and Contact Heat Evoked Potential Aδ amplitudes after leg stimulation.

PGP 9.5 and TRPV1 fibre density showed significant positive correlations with leg evoked potential Aδ amplitudes (Figure 6; PGP 9.5: r_s _= 0.51, p = 0.0006, TRPV1: r_s _= 0.48, p = 0.0012). When patients with absent evoked potentials were excluded from the analysis of correlation with PGP 9.5 nerve fibre density, the correlation was also significant (r_s _= 0.67, p = 0.004); however, this latter correlation was not significant for TRPV1 (r_s _= 0.23, p = 0.36). Correlation of arm evoked potentials Aδ amplitudes and nerve fibre density in the calf was also significant when all patients were included in the analysis (PGP 9.5, r_s _= 0.46, p = 0.002; TRPV1, r_s _= 0.35, p = 0.024).

An overview of sensory small fibre test results from the leg and correlations between tests is shown in Table 1.

**Table 1:** Overview of sensory small fibre tests and correlations. All data presented from the leg.

		**Patients**	**Controls**	**Correlations**
**Contact heat evoked potentials**	Latency (ms)	509 ± 0.015	525 ± 0.017	-
	Amplitude (μV)	3.63 ± 0.85	11.7 ± 1.95	• PGP 9.5 and TRPV1 IEF • Flare area
**Skin biopsy (IEF/mm)**	PGP 9.5	3.76 ± 0.48	6.52 ± 1.43	• Flare area • Evoked potential amplitude
	TRPV1	3.45 ± 0.52	5.7 ± 1.4	• Evoked potential amplitude
**Skin flare**	Area (cm^2^)	12.03 ± 0.90	18.14 ± 2.28	• PGP 9.5 IEF • Evoked potential amplitude
**QST (°C change)**	Warm	8.2 ± 0.8	6.3 ± 0.3	-
	Heat	13.97 ± 0.6	10.1 ± 0.2	-
	Cool	3.6 ± 0.6	2.1 ± 0.1	-

## Discussion

Objective measures of nociceptor fibre loss or dysfunction can serve as important tools to help diagnose sensory small fibre neuropathy, and to distinguish neuropathy from other chronic pain states. These may also potentially serve as indicators of analgesic efficacy. The Contact Heat Evoked Potential Stimulator (CHEPS) can be used to stimulate a range of heat sensitive receptors expressed by Aδ and C fibres, and the resulting evoked potentials can been recorded and measured [1]. To the best of our knowledge, this is the first time that the use of CHEPS has been described in patients with small fibre neuropathy. Contact heat evoked potentials recorded from patients with small fibre sensory neuropathy were of substantially reduced Aδ amplitude from the face, arm and leg, in comparison with controls.

In this study we have focused on Aδ amplitudes as they provided the most robust signal; C fibre traces were also obtained but were less robust. Aδ amplitudes for our controls were slightly lower than those recorded by Granovsky et al (2005), however, we have used a more proximal site for thermode placement (i.e. volar forearm arm, compared to thenar eminence or dorsum of hand).

The apparent abnormalities of small sensory fibre function in our patients observed with the contact heat evoked potentials measurements were validated by demonstrating correlations with other objective measures – skin IEF fibre counts and histamine-induced flares. Reduction of PGP 9.5 and TRPV1 IEF showed a significant correlation with leg evoked Aδ potential amplitudes. A significant positive correlation was also demonstrated between skin flare area and leg evoked potential Aδ amplitude. CHEPS appears to be a sensitive measure, detecting abnormalities in some patients with clinical symptoms, but who are within normal limits for other tests, including thermal thresholds – while it may be speculated that the decrease or absence of contact heat evoked potentials in such patients may indicate early small fibre dysfunction (possibly due to a mechanism other than axonal degeneration – such as a sensory ion channelopathy), further studies are necessary to explore and confirm these possibilities. Skin biopsies and flares have the advantage over CHEPS and QST in that they localise the pathology within the peripheral nervous system. QST may reveal additional phenomena such as paradoxical sensations (burning sensation on cooling). It should also be noted that subsets of small fibres may be affected independently in small fibre neuropathies [19], which reflects the heterogeneous aetiology of the condition. The use of a full array of tests to assess and localise small fibre dysfunction is thus desirable, including QST, skin biopsies, skin flares and CHEPS, for the diagnosis of the condition, and to advance clinico-pathological correlations.

Skin biopsy is a sensitive method for the quantitative assessment of small sensory fibres, and has been considered the "gold standard" for diagnosing small fibre neuropathy [11-13,20,21]. The European Federation of Neurological Sciences (EFNS) guidelines commend skin biopsy for this diagnosis in preference to sural nerve biopsy [22]. However, analysis of skin biopsy is technically challenging for some investigators; the biopsy leaves a small scar and has a risk, albeit low, of complications, for example infection or keloid formation. Skin biopsies are avoided over some affected regions e.g. the sole, or palm. Our skin biopsy results compare favourably to others utilising skin biopsy with the same tissue section thickness [13].

Epidermal thickness in biopsies taken from our patients was significantly reduced compared to biopsies taken from controls, in keeping with a diagnosis of neuropathy. It is well known that denervation of the skin causes thinning of the epidermis and this has been illustrated experimentally in rat [23-25] and mouse models [26]. Swellings of nerve fibres and their varicosities were rarely observed in our patient cohort. We noted marked swellings on both large and fine calibre nerve fibres in only one patient in our study. This may reflect the stable symptomatology in most of our patients. The low detection frequency of swellings in our cohort of patients may alternatively reflect our routine use of 10 μm tissue sections, although some of our studies using thicker (50 μm) sections revealed no increase in the number of patients with this morphological phenomenon. We have seen higher frequencies of axonal swellings in diagnostic skin biopsies from patients with more acute neuropathies, in accord with other reports. Nerve fibre swellings have been reported previously in rodents and humans [21,27-29]. However, the significance of these morphological changes is uncertain [30]. Some studies suggest that they predict nerve degeneration [23,31], and mark the activity of the neuropathic process or the pre-degeneration of nociceptive fibres. In addition, they have been correlated with the early development of neuropathic symptoms, abnormal heat pain thresholds [32], and a decline in the density of epidermal nerve fibres [33]. Other studies have shown that swellings are found in regenerated fibres after skin blister or capsaicin denervation, and have been associated with improved sensation and re-innervation [30,34].

Previous work on the axon reflex flare has shown that C-nociceptor fibre activation is the main factor determining its area [35]; the correlation with CHEPS responses suggests parallel pathology in Aδ and C fibres in our patients. Flares are known to be diminished in patients with small fibre neuropathies [8-10]. Recently, axon reflex flares have been shown to represent a potential biomarker for TRPV1 antagonists: a reduction of heat-evoked pain and capsaicin-evoked flare area by the TRPV1 antagonist SB705498 was demonstrated in a study of 19 healthy human volunteers [36]. CHEPS can be used in areas unsuitable for histamine-induced flares such as the face or glabrous skin. We have previously reported correlations of nerve growth factor (NGF), which regulates TRPV1 expression, with skin flare area in patients with diabetic neuropathy [14,37].

The EFNS guidelines on neuropathic pain assessment [22] recommend laser evoked potentials as a reliable method of assessing nociceptive pathways, and of diagnostic use in peripheral neuropathy. They have been used successfully in studies of patients with peripheral neuropathies [38], trigeminal neuralgia [39], post herpetic neuralgia [40], and syringomyelia [41]. Laser evoked potential suppression helps diagnose neuropathic pain states [39,42,43], while laser evoked potential facilitation is described in fibromyalgic and chronic inflammatory pain [44,45]. The similarity between contact heat evoked and laser evoked potentials has already been described [5]. Lower amplitudes of laser evoked potentials were reported following stimulation of the trigeminal nerve in diabetic patients [46], with a number of absent responses in affected patients, as in our study with contact heat evoked potentials. Similar changes in laser evoked potentials were also reported following stimulation of the feet in diabetic patients [47,48] with no clinical or electrophysiological evidence of large fibre dysfunction. CHEPS thus appears to produce similar cerebral evoked nociceptor potentials to laser stimulation in patients, at least in the regions we have tested. In comparison with lasers, the CHEPS system is easy to operate and calibrate, and it allows for repetitive stimulation or "wind-up", avoiding any risk of superficial burns. However, CHEPS does involve contact with the skin, which may be uncomfortable in some patients with allodynia, and, in principle, the skin contact may affect pathways not activated by heat or noxious stimuli. A discussion comparing thermal conduction and thermal radiation in the study of the nociceptive system has been conducted [49,50]. Conductive heat offers the advantage of control over temperature at the thermode-skin interface (via a thermocouple in the stimulator), and while lasers avoid the simultaneous stimulation of low threshold mechanoreceptors, they can cause variations in baseline temperatures. Also discussed, was the problem of estimating the activation temperature at the nerve receptor level with the CHEPS system. Heat is dissipated at the skin-thermode due to a heat sink effect, which is not seen with modern lasers, where varying the wavelength can change the depth of penetration.

CHEPS could potentially be used as a valuable tool in future trials of novel therapeutic agents in experimental pain models, as contact heat evoked potentials may help monitor the effects of analgesic intervention. It may be particularly useful in the development of TRPV1 antagonists for chronic pain states. TRPV1, a member of the vanilloid receptor family localizes mainly to small sensory fibres, and is a ligand-gated ion channel activated by vanilloids, noxious heat and protons. The association between TPRV1 immunoreactivity and tissue hypersensitivity has been demonstrated for a number of hypersensitivity states in humans [18,51]. TRPV1 antagonists have been shown to relieve pain in rodent models [52], and clinical trials are underway.

## Conclusion

In summary, we have shown that the contact heat evoked potential stimulator (CHEPS) can provide a practical, rapid and non-invasive additional clinical tool of potential utility in the evaluation of small fibre neuropathy and neuropathic pain states. Further evaluation of the technique is underway to identify a potential role in analgesic development.

## Competing interests

The author(s) declare that they have no competing interests.

## Authors' contributions

DDA was involved in patient testing, clinical assessment, data analysis and writing the manuscript. PF performed the immunohistochemistry, helped with data analysis and writing the manuscript. KMR helped with patient testing, immunohistochemistry, data analysis and writing the manuscript. VPM performed nerve conduction studies in some patients, participated in the conception of the study and helped to interpret the data. BAC and CB helped conceive the study, interpret the data and write the manuscript. PA conceived the study and participated in its design and co-ordination, interpretation, and completion of the manuscript. All authors read and approved the manuscript.

## Pre-publication history

The pre-publication history for this paper can be accessed here:


